# Performance-Enhancing Methods for Au Film over Nanosphere Surface-Enhanced Raman Scattering Substrate and Melamine Detection Application

**DOI:** 10.1371/journal.pone.0097976

**Published:** 2014-06-02

**Authors:** Jun Feng Wang, Xue Zhong Wu, Rui Xiao, Pei Tao Dong, Chao Guang Wang

**Affiliations:** 1 College of Mechatronics and Automation, National University of Defense Technology, Changsha, Hunan, P. R. China; 2 Beijing Institute of Radiation Medicine, Beijing, P. R. China; 3 State Key Laboratory of Transducer Technology, Chinese Academy of Science, Shanghai, P. R. China; Queen’s University Belfast, United Kingdom

## Abstract

A new high-performance surface-enhanced Raman scattering (SERS) substrate with extremely high SERS activity was produced. This SERS substrate combines the advantages of Au film over nanosphere (AuFON) substrate and Ag nanoparticles (AgNPs). A three order enhancement of SERS was observed when Rhodamine 6G (R6G) was used as a probe molecule to compare the SERS effects of the new substrate and commonly used AuFON substrate. These new SERS substrates can detect R6G down to 1 nM. The new substrate was also utilized to detect melamine, and the limit of detection (LOD) is 1 ppb. A linear relationship was also observed between the SERS intensity at Raman peak 682 cm^−1^ and the logarithm of melamine concentrations ranging from 10 ppm to 1 ppb. This ultrasensitive SERS substrate is a promising tool for detecting trace chemical molecules because of its simple and effective fabrication procedure, high sensitivity and high reproducibility of the SERS effect.

## Introduction

Surface-enhanced Raman scattering (SERS) spectroscopy is an important analytical technique for biological sensing and trace analysis because of the enormous Raman enhancement for molecules absorbed on specific SERS substrates. Given that single molecule detection [1] has been achieved by means of SERS, extensive research has been carried out and several good review articles [2]–[4] have been published on this topic. Considering its high sensitivity and finger-printing capability, SERS has been applied in various fields, particularly in the trace detection of DNA [5]–[7], pesticide residues [8], [9], heavy metal ions [10] and other chemical molecules such as trinitrotoluene [11] and melamine [12]–[14].

Researchers have produced many SERS substrates with high SERS activity, among which metal film over nanosphere (MFON) [2], [15], [16] and metallic nanoparticles (MNPs) in suspension [17], [18] are widely utilized. Au film over nanosphere (AuFON) and Ag film over nanosphere (AgFON) are the two commonly used MFON SERS substrates easily produced by nanosphere lithography techniques [19], [20] that use spin-coated microspheres layers as templates for silver or gold deposition. The advantages of these MFON substrates are the high controllability of their relatively simple fabrication process and the high reproducibility of the SERS effect. Further applications are limited by their relatively low enhancement effect. Metallic nanoparticles, especially Ag nanoparticles (AgNPs), play an important role in SERS because of their ease of preparation and relatively high enhancement. However, it suffers from the poor colloidal stability and the poor reproducibility of the SERS effect.

In this paper, a simple and effective process is proposed to prepare a new high performance SERS substrate (AgNPs/AuFON) that combines the advantages of the AuFON substrate and AgNPs. First, we achieved the preparation and characterization of the SERS substrate. Nanosphere lithography techniques [19], [20] and the microwave synthesis method [21], [22] were used to fabricate the AuFON substrate and silver nanoparticles respectively. Through a simple amination process, the new AgNPs/AuFON SERS substrate was prepared. These fabrication processes were characterized by a field emission scanning electron microscope (FE-SEM), a transmission electron microscope (TEM) and a UV-vis spectrometer. Second, Rhodamine 6G (R6G) was used as a probe molecule to evaluate the SERS performance of the new SERS substrate. Finally, the detection of trace amounts of melamine on the AgNPs/AuFON SERS substrate was achieved.

## Experimental

### 1. Materials

All chemicals were of analytical grade and were utilized as received, unless mentioned otherwise. PS nanospheres with mean diameters of 509 nm purchased from Sphere Scientific Corporation (Wuhan) were utilized for the preparation of the AuFON SERS substrate. Three-inch silicon wafers were used as substrates. Gold pellets (99.999% purity) and chromium pellets (99.99% purity) were purchased from Jinyu Aochen (Beijing). Rhodamine 6G (R6G), 3-Aminopropyltriethoxysilane (APTES), and melamine were purchased from Sigma-Aldrich. Silver nitrate, sodium citrate, ethanol, acetic acid, and 30% hydrogen peroxide solution were purchased from Sinopharm Chemical Reagents (Shanghai). All aqueous solutions were made with Millipore ultrapure water (purified with Milli-Q system, 18.2 MΩ cm^−1^).

### 2. AuFON Substrate Fabrication

The fabrication procedure includes two parts: the preparation of the PS monolayer colloidal crystal and metal film deposition.

The PS monolayer colloidal crystal was fabricated by spin-coating technology based on our previous work [23]. First, the silicon wafer was ultrasonically cleaned in acetone, ethanol, and de-ionized (DI) water individually for half an hour, and then treated with piranha solution (3∶1 H_2_SO_4_/30% H_2_O_2_) at 80°C for 1 hour to raise the surface hydrophilicity. Second, the PS nanosphere suspensions (10%, w/v) were dropped on the silicon substrate. The spin-coating process started after 15 s of wetting and the final rotation speed of 1850 rpm was reached in approximately 2 s. After 180 s of spin-coating, the monolayer colloidal crystal film was formed.

Metal films were deposited in a vacuum deposition system (ZZS500, Chengdu Nanguang). A 10 nm Cr layer was first deposited on the PS monolayer film, and then 230 nm of Au was deposited with a chamber pressure of 10^−4^ Pa. The deposition rate for each film (5 Å/s) was monitored by a quartz crystal microbalance. After the metal film deposition, we accomplished the AuFON substrate fabrication.

### 3. Synthesis of Silver Colloids

The microwave synthesis method [21], [22] was used for the synthesis of silver colloids. First, silver nitrate (37 mg) was suspended in 100 mL ultrapure water, and then 2.6 mL 1% sodium citrate solution was added. After vigorous stirring, the mixed solution was placed in a microwave oven. After several minutes of boiling under high-fire (∼600 W), the conical flask was removed from the oven and cooled at Room Temperature (RT). After cooling, we adjusted the volume to 100 mL with ultrapure water to ensure consistency between several experiments. The synthesized silver colloids were investigated by means of TEM and UV-vis absorption spectrum. When measuring the UV-vis absorption spectrum, the silver colloids were diluted 100 times with ultrapure water.

### 4. Preparation of the AgNPs/AuFON SERS Substrate

The fabrication procedure of the active AgNPs/AuFON SERS substrate was relatively simple. First, the AuFON substrate was treated with 30% H_2_O_2_ instead of piranha solution for 1 h to protect the AuFON substrate from being destroyed. After the surface treatment, the AuFON substrate was thoroughly rinsed with ethanol and ultrapure water individually, and then the surface was aminated by dipping into a 1000∶10∶1 (v/v/v) ethanol/APTES/acetic acid solution for 1 h. After drying at RT, the modified AuFON substrate was cut into 3 mm×3 mm pieces. Second, 10 µL of silver colloids was dropped onto each piece of the AuFON substrate, followed by 5 µL 0.1% NaCl. After drying at RT, the active AgNPs/AuFON SERS substrate was successfully fabricated.

The SERS substrate was immersed in 100 µL analyte solution of different concentrations for 1 h before the SERS characterization to assure saturated adsorption.

### 5. Instrumentation

FE-SEM micrographs were acquired using a JEOL JSM-7001F FE-SEM operating at 5 kV. TEM images were taken using a Hitachi H-7650 TEM operating at 80 kV. UV-vis spectra were obtained using a Shimadzu UV-2600 spectrometer. Raman spectra were obtained using a portable Raman system B&W Tek, i-Raman Plus BWS465-785H spectrometer. The 785 nm laser was utilized as the excitation source. Its maximum laser power was 340 mW at excitation port, however, the maximum laser power on the sample was actually around 275 mW due to laser transmission loss, which was measured by a power meter (Coherent, lasercheck). The light from the laser was focused on a sample via a 20× microscope objective. The laser beam spot size was around 105 µm with the working distance at 8.8 mm. The back-illuminated CCD cooled at −2°C was used as the detector.

## Results and Discussion

### 1. Fabrication Process of the AuFON Substrate

**Figure 1:**
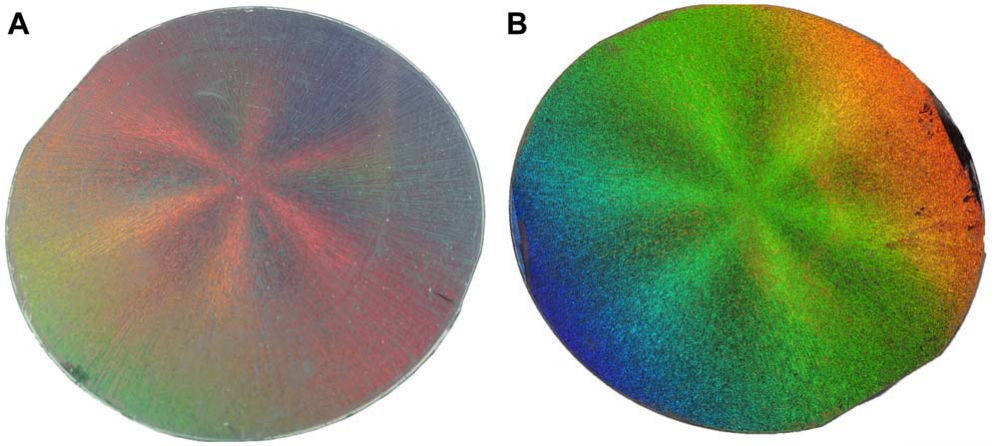
Six-beam diffraction pattern formed on a 3-inch wafer. A: PS colloidal crystal; B: AuFON.

A digital camera and FE-SEM were utilized to obtain the macroscopic and microcosmic images, respectively, to monitor the formation of AuFON substrate. Optically, PS colloidal crystals can produce Bragg diffraction of light in the optical region [24]. When illuminated with white light, the PS colloidal crystals before and after metal film deposition can both exhibit distinctive brilliant, six-beam diffraction with exactly 60 degrees between neighboring arms easily recognizable by the naked eyes. A digital camera was utilized to obtain the macroscopic images of the six-beam diffraction pattern formed on the 3-inch wafer, as shown in Fig. 1. These diffraction patterns indicate the long-range order of PS colloidal arrays [23].

After the metal film deposition on the self-assembled PS monolayer colloidal crystal, we obtained the AuFON SERS substrate. From Fig. 1, the AuFON substrate can exhibit brighter colors than the PS colloidal crystal under sunlight. The surface color difference is related to the angle from which the photo was taken.

**Figure 2:**
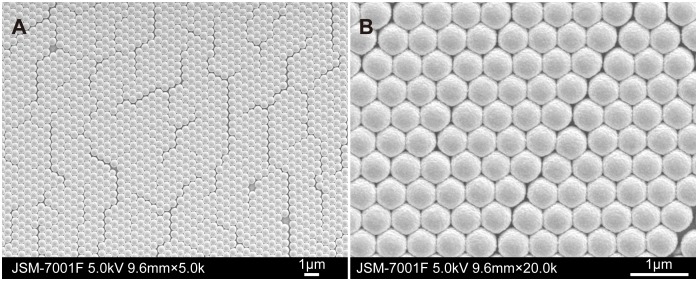
Low and high magnification FE-SEM images of the AuFON substrate. A: 5.0 k; B: 20.0 k.

FE-SEM was used to study the AuFON substrate in more details (Fig. 2). Fig. 2A shows the long-range order of the PS colloidal arrays, as depicted in Fig. 1. The rough gold surface, which is essential to SERS, can be easily observed in Fig. 2B.

### 2. Characteristics of Silver Colloids

**Figure 3:**
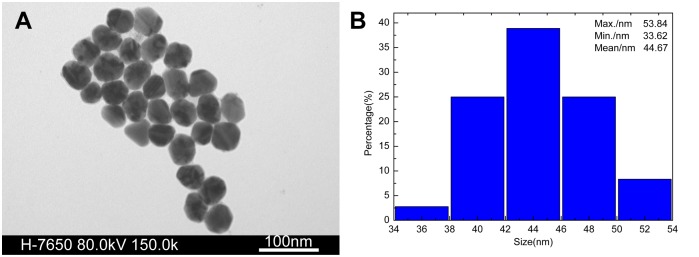
TEM images and size distribution of AgNPs. A: TEM image under 150.0 k; B: size distribution of AgNPs.

TEM and UV-vis absorption spectrum were used to investigate the particle morphology of the synthesized Ag colloids. The sample was prepared utilizing the procedure described above. The TEM images and the size distribution of AgNPs prepared in this work are shown in Fig. 3. Most of these AgNPs are spherical in shape. The nanoparticles’ sizes are relatively homogeneous, mainly distributing from 34 nm to 54 nm as shown in Fig. 3. The mean diameter is about 45 nm.

**Figure 4:**
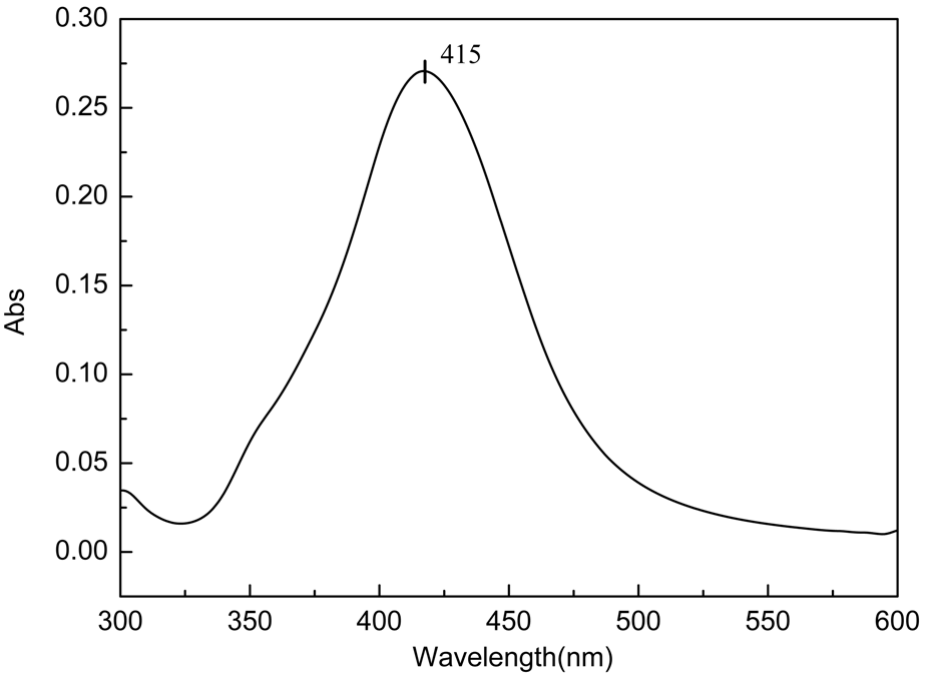
UV-vis spectra of the silver colloidal solution.

The synthesized Ag colloids were also investigated by UV-vis spectrum, as shown in Fig. 4. When measuring the UV-vis spectrum, a 10 µL Ag colloidal solution was added to 990 µL of ultrapure water to dilute by 100 times. From Fig. 4, the prepared sample exhibits a very distinct surface plasmon absorption band at approximately 415 nm. The symmetry of this curve also indicates that the AgNPs are roughly spherical in shape and have a narrow size distribution, which is consistent with the TEM images.

### 3. Characteristics of AgNPs/AuFON SERS Substrate

**Figure 5:**
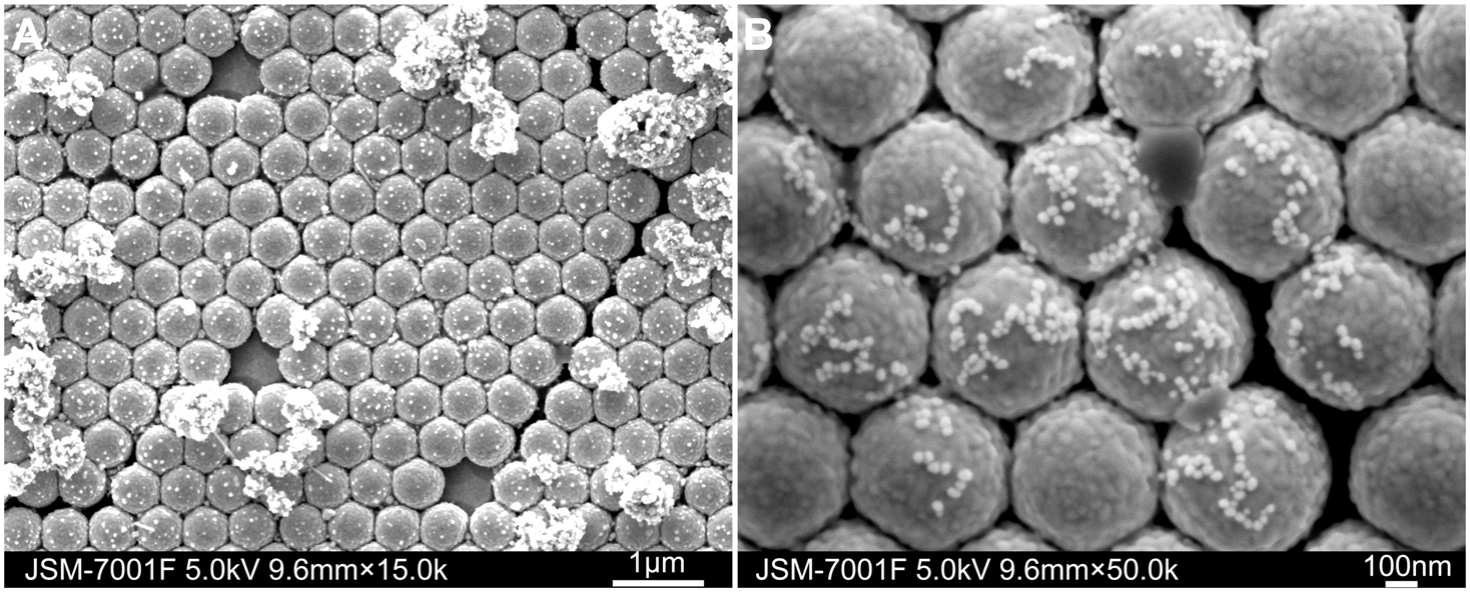
FE-SEM images of the AgNPs/AuFON substrate. A: 15.0 k; B: 50.0 k.

FE-SEM was used to monitor the formation of the AgNPs/AuFON SERS substrate. The prepared new SERS substrate is shown in Fig. 5. Comparing with the AuFON substrate, the new substrate is different mainly in two aspects. The first aspect is the surface modification of AuFON, as shown in Fig. 5B. After the surface amino treatment, the exposed amino groups can capture colloidal silver particles. The other different aspect is the slight aggregation of colloidal silver particles on AuFON, as shown in Fig. 5A. These aspects are the two main reasons for the performance enhancements of the AgNPs/AuFON substrate.

### 4. SERS Spectra of R6G on the AuFON and AgNPs/AuFON SERS Substrates

R6G was used as a probe molecule to evaluate the SERS performance of these two different SERS substrates because of its large cross section and well-characterized Raman bands [25]. When evaluating the SERS performance using R6G, we collected five spectra from different sites from each sample and averaged to represent the SERS results.

**Figure 6:**
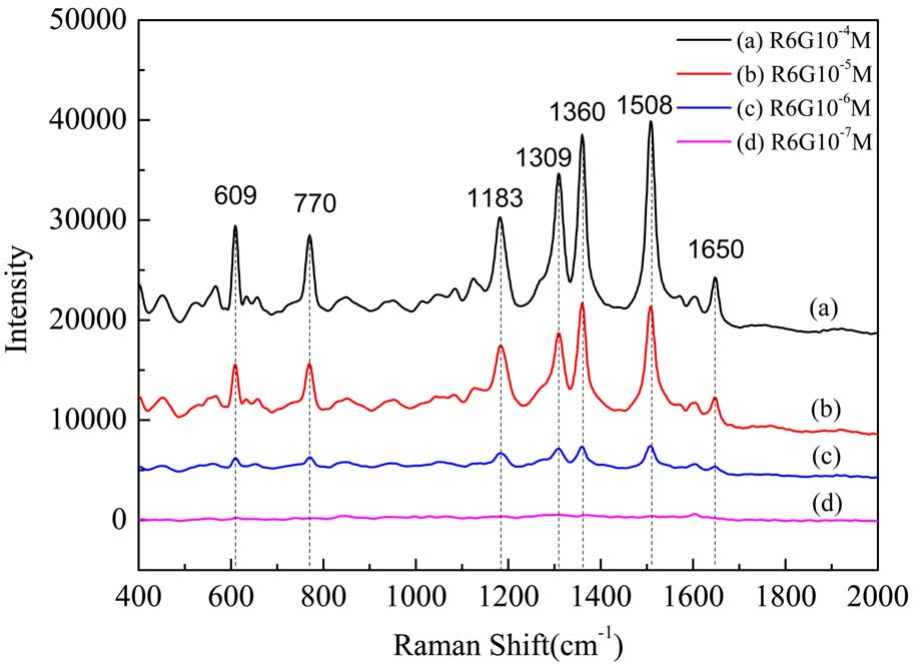
SERS spectra of R6G with different concentrations on the AuFON substrate. A: 10^−4^ M; B: 10^−5^ M; C: 10^−6^ M; D: 10^−7^ M.

First, the SERS spectra of R6G with different concentrations on the AuFON substrate were investigated. Back-illuminated CCD was used as the detector, the accumulation time was 5 s, and the incident power was 10% of the laser excitation power. In the present experiments, SERS spectra of 10^−4^, 10^−5^, 10^−6^ and 10^−7^ M R6G on the AuFON substrate were obtained. The detection limit for the AuFON substrate prepared by our laboratory could reach 10^−6^ M, as shown in Fig. 6. Several strong Raman bands at 1650, 1508, 1360, 1309, 1183, 770 and 609 cm^−1^ are observed for 10^−4^, 10^−5^, and 10^−6^ M R6G. These Raman bands observed by our experiments are very consistent with other researcher works [25] and the fluctuation is limited to 5 cm^−1^, as shown in Table 1.

**Table 1:** Raman bands of the R6G molecule on the AuFON substrate.

Current paper (cm^−1^)	Hildebrandt et al. [25] (cm^−1^)	Vibration mode [25]
1650	1650	arom C-C str
1508	1509	arom C-C str
1360	1363	arom C-C str
1309	1310	
1183	1181	C-C str
770	773	C-H op bend
609	614	C-C-C ring

Note: arom = aromatic ring; str = stretching; op = out-of-plane; bend = bending.

However, the AuFON substrate was unable to detect the 10^−7^ M R6G. In this paper, we propose a simple method to enhance the AuFON SERS substrate’s performance. AgNPs are easy to fabricate through regular wet chemistry [17], providing many choices in terms of size and shape. By aggregating the AgNPs from their suspensions, this type SERS substrate can achieve numerous hot spots and lead to enormous enhancements in realizing single molecule detection [1]. However, its application is limited because of its reproducibility problem. The reproducibility problem can be mitigated by fixing the AgNPs on a type of solid support [3]. In the present study, we utilized silver colloids to enhance the SERS performance of the AuFON substrate.

**Figure 7:**
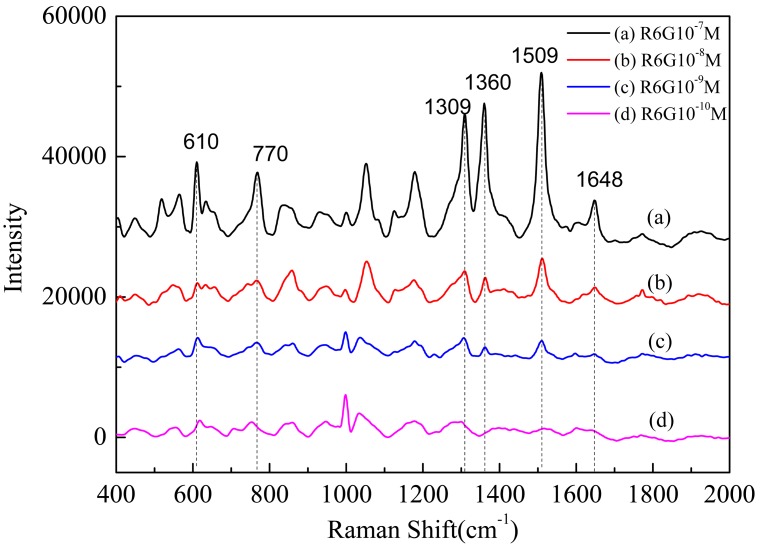
SERS spectra of R6G with different concentrations on the AgNPs/AuFON substrate. A: 10^−7^ M; B: 10^−8^ M; C: 10^−9^ M; D: 10^−10^ M.

Second, we studied the SERS spectra of R6G with different concentrations on the AgNPs/AuFON substrate. When measuring Raman spectra, the accumulation time was set at 50 s, and the incident power at 10% of the laser power. SERS spectra of 10^−7^, 10^−8^, 10^−9^, and 10^−10^ M R6G on the AgNPs/AuFON substrate were obtained, as shown in Fig. 7. By comparing Fig. 6 and Fig. 7, it can be easily found that the detection limit of new SERS substrate was improved approximately by 3 orders comparing with the AuFON substrate fabricated by our laboratory. Similar Raman bands for 10^−7^, 10^−8^ and 10^−9^ M R6G were also observed, which were consistent with previous experiments, as shown in Fig. 7.

### 5. Melamine Detection Application

Melamine has attracted wide attention since the U.S. pet food contamination incident in 2007. In September 2008, China experienced the contaminated Sanlu infant milk powder incident, resulting in infants who consumed the contaminated milk powder to suffer from kidney stone disease. This incident is also related to melamine. In November 2008, the safety/risk assessment of the US Food and Drug Administration (FDA) concluded that levels of melamine and its analogues below 2.5 ppm in foods other than infant formula do not raise public health concerns.

In this paper, melamine was selected to demonstrate the practicality of this new AgNPs/AuFON SERS substrate. A 10^3 ^ppm melamine solution was prepared as a stock solution. It was serially diluted in ultrapure water to prepare samples down to 0.1 ppb.

**Figure 8:**
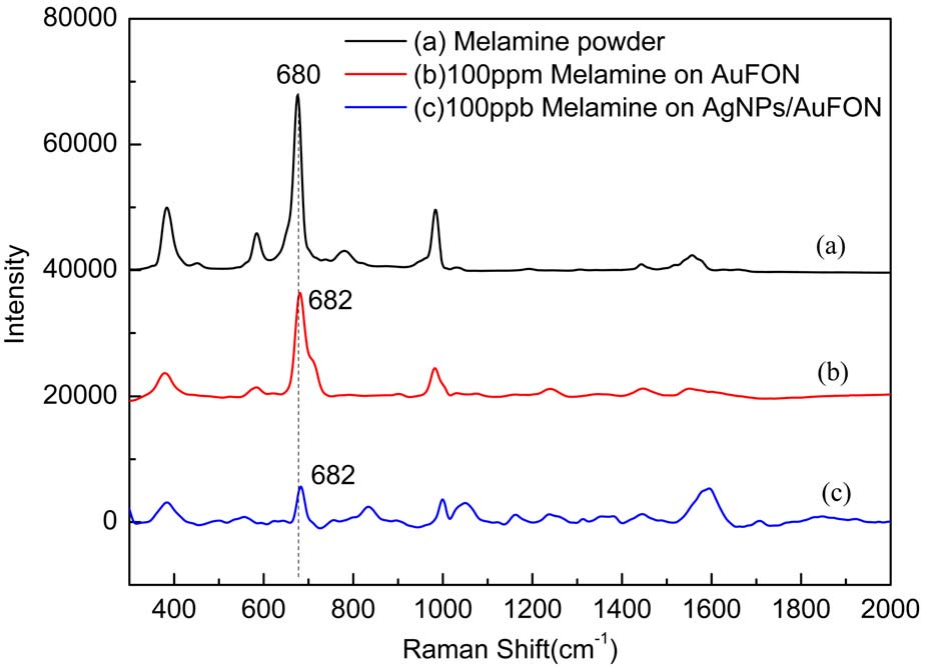
SERS spectra of melamine on different SERS substrates. A: melamine powder on slide; B: 100 ppm melamine on AuFON; C: 100 ppb melamine on AgNPs/AuFON.

SERS spectra of melamine on different SERS substrates were recorded to verify the performance-enhancing effect of the AgNPs/AuFON substrate (Fig. 8). The accumulation time was set at 20 s and the incident power at 20% of the laser power when measuring the Raman spectra. From Fig. 8, the main Raman peak of melamine at approximately 682 cm^−1^, which belongs to the in-plane deformation vibration of the triazing ring [12], can be easily observed in all three curves. The SERS spectra of melamine powder on slide are a little different from the spectra of melamine solutions on SERS substrates because of the detection condition difference with a tolerable fluctuation of 2 cm^−1^, as shown in Fig. 8. It can be roughly estimated that the SERS performance of the AgNPs/AuFON substrate is enhanced by 3 orders compared with AuFON from this figure.

**Figure 9:**
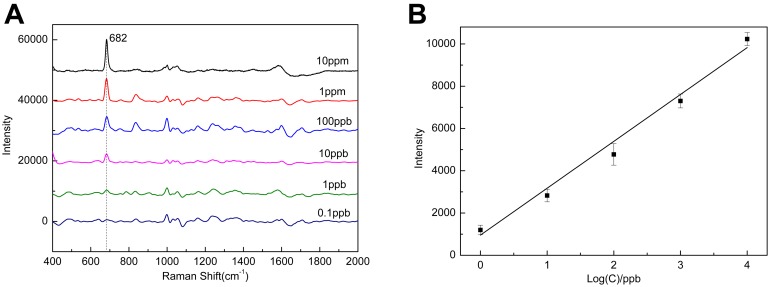
Melamine detection application. A: SERS spectra of melamine on the AgNPs/AuFON; B: SERS intensity at 682 cm^−1^ vs logarithm of the concentrations.

A series of melamine solutions ranging from 1 ppm to 0.1 ppb were prepared to demonstrate the practicality of this new AgNPs/AuFON SERS substrate in more detail. The SERS spectra were recorded as shown in Fig. 9A. The LOD of melamine is 1 ppb, which is far lower than the FDA standard issued in 2008. We conducted 15 tests for each of the samples and made a statistical analysis of the results obtaining the intensity-concentration curve, as shown in Fig. 9B. A linear relationship is observed from 1 ppb to 10 ppm and the correlation coefficient is calculated to be 0.9868.

## Conclusions

In this study, a new high performance SERS substrate AgNPs/AuFON was successfully prepared. First, we achieved the preparation of AuFON by nanosphere lithography techniques and the synthesis of silver colloidal by the microwave synthesis method. The new AgNPs/AuFON SERS substrate was then prepared through a simple amination process. These fabrication processes were characterized by FE-SEM, TEM, and a UV-vis spectrometer. Second, R6G was used as a probe molecule to evaluate the SERS performance of the new SERS substrate. A 3-order enhancement of SERS was observed compared with the SERS effect between the new substrate and AuFON. Finally, the detection of trace amounts of melamine on the AgNPs/AuFON SERS substrate was achieved, and the detection limit was 1 ppb.
